# The Role of the Cardioversion Defibrillator in Post Myocardial Infarction Sudden Cardiac Death: A Systematic Review of Clinical Trials and Observational Studies

**DOI:** 10.7759/cureus.4314

**Published:** 2019-03-25

**Authors:** Muhammad Haisum Maqsood, Kinza Rubab

**Affiliations:** 1 Internal Medicine, King Edward Medical University / Mayo Hospital, Lahore, PAK

**Keywords:** sudden cardiac death, cardioversion, defibrillator

## Abstract

Sudden cardiac death (SCD) accounts for approximately half of all the deaths attributed to cardiovascular disease in the United States. Survivors of an acute myocardial infarction (AMI) are at high risk of SCD, largely due to cardiac arrhythmias and severe left ventricular (LV) systolic dysfunction. The implantable cardioverter defibrillator (ICD) or automated implantable cardioverter defibrillator (AICD) is a device that is implantable inside the body, able to perform cardioversion, defibrillation, and (in modern versions) pacing of the heart. According to a study included in our review, patients who received an ICD contributed to an adjusted 44% reduction (hazard ratio [HR] 0.56, 95% CI: 0.32-1.01; P = 0.053) of all-cause mortality compared to those with a comparable baseline. Patients with an ICD implant three months after a myocardial infarction (MI) demonstrated a non-significantly higher mortality than patients who did not receive an ICD. The factors favoring ICD implantation were multiple MIs, increased resting heart rate, occurrence of non-sustained ventricular tachycardia, QRS duration = 120 ms, syncope events, anti-arrhythmic drug treatment (mostly Class III), and an index MI of more than one year. The likelihood of receiving an ICD diminished with the patient’s age. Increased periodic repolarization dynamics were a significant predictor of mortality. It can be concluded that cardioverter defibrillators help reduce not only all-cause mortality but also sudden cardiac death. It is important to note that ICDs are only significant if implanted after a sufficient time-gap post-MI.

## Introduction and background

Description of the condition

Sudden cardiac death (SCD) is a significant public health problem and accounts for approximately half of all the deaths attributed to cardiovascular disease in the United States [1]. The incidence of SCD ranges from 0.36 to 1.28 per 1,000 persons per year with approximately 400,000 deaths occurring annually in the United States alone [2-5]. It is most commonly associated with coronary artery disease and can be its initial manifestation or may occur in the period after an acute myocardial infarction [6]. Survivors of an acute myocardial infarction (AMI) are at high risk of SCD, largely due to cardiac arrhythmias and severe left ventricular (LV) systolic dysfunction [7]. Several studies have shown that about 5% to 10% of patients suffer SCD within the first year after the acute event [8- 11]. Despite improvements in the treatment of coronary artery disease, more than half of all cardiovascular mortality is due to arrhythmic death [12]. Purkinje fibers, surviving but dysfunctional, are the main arrhythmogenic foci during the infarct evolution period. After one hour of ischemia, the Purkinje and surviving muscle fibers exhibit reduced resting potentials, decreased action potential amplitudes, and reduced upstroke velocities. The action potential duration, prolonged in muscle fibers, is shortened in Purkinje fibers, facilitating re-entry. Re-entry, flow of injury current, and abnormal automaticity (delayed after-depolarization) lead to arrhythmias [13].

Description of the intervention

Decreasing the rate of sudden cardiac death requires the identification and treatment of at-risk patients through evidence-based pharmacotherapy and interventional strategies aimed at primary and secondary prevention [14]. With the introduction of modern treatment, the incidence of SCD in myocardial infarction (MI) survivors may decline in parallel with coronary heart disease, especially in patients receiving optimal medical therapy (ß-blockers, aspirin, statins, and angiotensin-converting enzyme [ACE] inhibitors) and revascularization [15-16]. Unfortunately, of all patients experiencing acute myocardial infarction (MI), usually in the form of ST-elevation MI, 25-35% die of SCD before receiving medical attention, most often from ventricular fibrillation. Prognosis is better for patients who come to the hospital on time. Reperfusion therapy, better attained with primary percutaneous coronary intervention than with thrombolysis, has made a big difference in reducing the risk of SCD early and late after ST-elevation MI. In-hospital SCD due to ventricular tachyarrhythmias is manageable with preventive measures, drugs, or electrical cardioversion [14].

The implantable cardioverter defibrillator (ICD) or automated implantable cardioverter defibrillator (AICD) is a device implantable inside the body and able to perform cardioversion, defibrillation, and (in modern versions) pacing of the heart. ICD has the capability to rectify most life taking cardiac arrhythmias. The ICD is the first-line treatment and prophylactic therapy for patients at risk for sudden cardiac death due to ventricular fibrillation and ventricular tachycardia [17]. Since their introduction in the 1980s, the evidence supporting the use of implantable cardioverter defibrillators (ICDs) has steadily increased. Implantable cardioverter defibrillators are now the primary treatment modality for the prevention of SCD in high-risk patients [18-19]. Several professional societies have issued guidelines for ICD therapy for the primary prevention of SCD post-myocardial infarction (post-MI) [20-23].

Why is it important to do this review?

The absence of reviews that have assessed the role of cardioversion defibrillation, particularly in post-MI sudden cardiac death, has prompted us to evaluate the available evidence to establish the benefits and harms of the cardioverter defibrillator in such cases.

Objective

To evaluate the role of cardioversion defibrillation in post-MI sudden cardiac death.

## Review

Descriptive analyses/findings

Effect on All-cause Mortality

According to a study included in our review, patients who received an ICD contributed to an adjusted 44% reduction (HR 0.56, 95% CI: 0.32-1.01; P = 0.053) of all-cause mortality compared to those with comparable baseline characteristics but without an ICD. All-cause mortality of ICD recipients was significantly lower if the ICD was implanted later than 11 months after an acute MI (P = 0.001) [24].

Effect on Sudden Cardiac Death

According to a study included in our review, ICD-therapy was associated with a reduction of SCD by 53.1% (P <0.001) [25].

Decision Criteria

According to a study that we have included, patients with a left ventricular ejection fraction (LVEF) less than 30% had a 31-fold chance of receiving an ICD; patients with a 30%-40% LVEF had a six-fold chance, compared with the group having an LVEF greater than 40% to receive an ICD (P = 0.001). Other factors favoring ICD implantation were multiple MIs, increased resting heart rate, the occurrence of non-sustained ventricular tachycardia, QRS complex duration = 120 ms, syncope events, anti-arrhythmic drug treatment (mostly Class III), and an index MI of more than one year. The likelihood of receiving an ICD was reduced with older patient age [24].

Time from Index MI

The studies we have included found that the ICD implant three months after MI demonstrated a non-significantly higher mortality than patients who did not receive an ICD (HR 2.1, 95% CI: 0.95-4.65; P = 0.068). A time period of 4-11 months after an MI showed that an ICD revealed a non-significant, moderate reduction of mortality (HR 0.72, 95%, CI: 0.29-1.78; P = 0.469), and a subgroup of patients with ICD implantation more than 11 months after their index MI showed a significantly reduced mortality (HR 0.14, 95% CI: 0.03-0.56; P = 0.006) [24].

Association with Periodic Repolarization Dynamics

Periodic repolarization dynamics (PRD) is a novel electrocardiographic phenomenon that refers to previously unknown oscillations of cardiac repolarization instability. Increased PRD was a significant predictor of mortality. Although increased PRD predicted SCD in conventionally treated patients (1.61 [1.23-2.11]; P <0.001), it was predictive of non-sudden cardiac death (N-SCD) (1.63 [1.28-2.09]; P <0.001) in ICD-treated patients. ICD treatment substantially reduced mortality in the lowest three PRD quartiles by 53% (P = 0.001). However, there was no effect in the highest PRD quartile (mortality increase by 29%; P = 0.412; P <0.001 for difference), because the reduction of N-SCD was compensated for by an increase of N-SCD [25]. 

Risk Factors

Increased risk for SCD is predicted by age, LVEF equal to or less than 25%, and non-revascularization [7].

Comparison with Revascularization

The results of a study included in this review showed that revascularization reduces the risk of SCD (2.7% compared to 9.6% in non-revascularization). Revascularization may reduce the risk of SCD in post-MI patients with an LVEF <35% on the basis of medical therapy [7].

Discussion

This systematic review has assessed the role of cardioversion defibrillation in post-MI sudden cardiac death. We included four studies and has revealed significant positive and negative aspects. As compared to the previous interventions, such as pharmacotherapy and revascularization, cardioversion defibrillation has a significant existence.

The increased risk for sudden cardiac death is predicted by age, LVEF equal to or less than 25%, and non-revascularization. Our systematic review signifies that the cardioverter defibrillator plays an important role in reducing all-cause mortality and sudden cardiac death. The reduction of SCD and mortality was more significant if an ICD was implanted after a longer time lapse from the index MI (particularly after 11 months). It also showed that the patients with an ejection fraction less than 40% had a greater probability of receiving an ICD than those with higher LVEF. Our study also involved periodic random depolarization, which is an important predictor of SCD in conventionally treated patients and that of negative SCD in ICD-treated patients.

The results of our systematic review are similar to the studies present in the literature. Our review endorses the renowned Multicenter Automatic Defibrillator Implantation Trial II (MADIT-II) that showed that prophylactic ICD reduces all-cause mortality by 31% in post-MI patients with systolic dysfunction (EF ≤30%), compared to standard medical therapy [19]. However, the Defibrillator in Acute Myocardial Infarction Trial (DINAMIT) showed that prophylactic implantation of an ICD 6-40 days after acute MI in patients with left ventricular (LV) dysfunction (LVEF ≤35%) and impaired autonomic function reduces arrhythmic deaths but does not improve all-cause mortality [26]. Similar to our results, the IRIS trial showed no overall survival benefit from starting implantable cardioverter defibrillator (ICD) therapy in the early weeks after an acute MI in patients with features that put them at increased risk for events [27]. The Multicenter Automatic Defibrillator Implantation Trial with Cardiac Resynchronization Therapy (MADIT-CRT) trial, conducted in the first decade of this century, stepped ahead and stated that among patients with heart failure (HF) with LVEF ≤30% and QRS complex duration ≥130 msec, placement of an ICD with cardiac resynchronization therapy reduces the rate of mortality or HF events, compared to ICD placement alone. This benefit was driven primarily by a reduction in HF events [19].

We revealed sufficient evidence regarding the impacts of the cardioverter defibrillator, particularly in post-MI sudden cardiac death. A drawback of the available literature is that it fails to evaluate the complications associated with these implants systematically. The limitations of the included studies did not enable us to derive conclusive evidence about the adverse outcomes, if any.

## Conclusions

It can be concluded that cardioverter defibrillators help reduce not only all-cause mortality but also sudden cardiac death. It is important to note that ICDs are only significant if implanted after a sufficient time-gap post-MI.
